# Considerable matrix shift in the electronic transitions of helium-solvated cesium dimer cation ${\text{Cs}}_{2}{\text{He}}_{n}^{+}$ †

**DOI:** 10.1039/c9cp04790e

**Published:** 2019-11-08

**Authors:** Lorenz Kranabetter, Nina K. Bersenkowitsch, Paul Martini, Michael Gatchell, Martin Kuhn, Felix Laimer, Arne Schiller, Martin K. Beyer, Milan Ončák, Paul Scheier

**Affiliations:** a Institut für Ionenphysik und Angewandte Physik, Universität Innsbruck, Technikerstr. 25, A-6020 Innsbruck, Austria; b Department of Physics, Stockholm University, 106 91 Stockholm, Sweden

## Abstract

We investigate the photodissociation of helium-solvated cesium dimer cations using action spectroscopy and quantum chemical calculations. The spectrum of Cs_2_He^+^ shows three distinct absorption bands into both bound and dissociative states. Upon solvation with further helium atoms, considerable shifts of the absorption bands are observed, exceeding 0.1 eV (850 cm^−1^) already for Cs_2_He_10_^+^, along with significant broadening. The shifts are highly sensitive to the character of the excited state. Our calculations show that helium atoms adsorb on the ends of Cs_2_^+^. The shifts are particularly pronounced if the excited state orbitals extend to the area occupied by the helium atoms. In this case, Pauli repulsion leads to a deformation of the excited state orbitals, resulting in the observed blue shift of the transition. Since the position of the weakly bound helium atoms is ill defined, Pauli repulsion also explains the broadening.

## Introduction

Vibrationally resolved electronic spectroscopy in combination with theoretical calculations is a powerful technique to determine details of the geometrical arrangement and electronic structure of molecules or clusters. The quality of vibrationally resolved spectra depends critically on the temperature, as recently demonstrated for the assignment of the structure of Au_4_^+^.^1^ This has triggered the development of several cryogenic techniques, *i.e*., molecular beams,^2,3^ matrix isolation,^4,5^ helium droplets,^6,7^ cryogenic traps^8–20^ and cryogenic storage rings.^21–25^ The low density of ionic targets requires special techniques, such as cavity ring down^26^ or action spectroscopy.^27,28^ Tagging of ions with a weakly boundmessenger turns out to be particularly suitable tomeasure absorption lines of ions, albeit leading to a matrix shift.^10,29^ The binding energy of helium to ions is lower than for any other atom or molecule, leading to a minimum matrix shift.^30^

Whereas only very few He atoms can be attached to singly-charged ions in cold traps, ions can be solvated with almost any number of He atoms when formed in doped helium nanodro-plets. In the case of C_60_^+^, a remarkable linear red-shift of the absorption lines for the first 32 He atoms attached could be used to extrapolate to the absorption line of the bare ion.^31–33^ In combination with theory, the experimental results of the matrix shift as well as the width of the absorption lines provide unprecedented insight into the details of the solvation of ions with helium, including phase transitions and isomeric effects. Alkali metal atoms reside on dimples at the surface of helium droplets, as shown in numerous experimental^21,34–40^ and theoretical studies.^41–46^ Alkali metal clusters, however, are submerged into the cluster above a critical cluster size.^46–48^ The groups of Ernst and Stienkemeier investigated photoionization of Cs atoms and the submersion of Cs^+^ into He droplets.^34,39^ Chen *et al*. managed to solvate Cs^+^ ions with several million helium atoms *via* pickup of Cs^+^ formed upon thermionic emission into large He droplets.^49^ Dopant ions ejected from charged helium droplets are sometimes complexed with a few helium atoms when recorded *via* mass spectrometry. The first solvation layer is particularly strongly bound, and its closure is typically reflected as a clear intensity drop of the ion series ${\text{MHe}}_{n}^{+}$ (with M^+^ the alkali metal ion) in mass spectra.^48,50–52^ Path integral or basin-hopping methods reproduce the experiments very well.^44,45^ It was also shown previously that helium adsorption might lead to appreciable spectral shifts.^53^

Spectroscopic properties of the Cs_2_^+^ ion were investigated previously in experimental^54,55^ and theoretical studies^56–58^ within the series of alkali metal dimers, assigning the lowest electronically excited states. In the present study, we explore photo-dissociation of ${\text{Cs}}_{2}{\text{He}}_{n}^{+}$ *via* action spectroscopy to assess helium solvation effects in a seemingly simple diatomic system. Single-reference and multi-reference *ab initio* calculations including spin–orbit coupling reproduce the experimental results and provide an assignment of the electronic transitions. A pronounced blue-shift of more than 0.1 eV (850 cm^−1^) for absorption into the $1^{2}{\text{Σ}}_{\text{u}}^{+}$ state for ${\text{Cs}}_{2}{\text{He}}_{n}^{+}$ with *n* = 10 provides clear evidence that the He atoms preferentially occupy positions along the axis of the cesium dimer cation.

## Methods

${\text{Cs}}_{m}{\text{He}}_{n}^{+}$ ions are formed upon electron irradiation (85 eV, 260 mA) of Cs doped He nanodroplets (average size about 10^5^ atoms, 2.7 MPa, 9.95 K expansion conditions). Penning ionization *via* electronically excited He* will be the dominant ionization channel for the heliophobic cesium atoms.^7,59–61^ Ionized cesium atoms and clusters have a substantially stronger interaction with the He matrix than their corresponding neutral precursors, thus leading to their submersion into the droplet.^46^ Low-mass ions ejected from the large multiply charged droplet are deflected by 90 degrees relative to the neutral beam *via* electro-static lenses. The beam is guided into the extraction region of a high-resolution time of flight mass spectrometer, where it is merged with a laser beam from a pulsed tuneable light source (EKSPLA NT 242, 210–2600 nm). Every tenth extraction pulse of the mass spectrometer operated at 10 kHz is irradiated with the laser (repetition rate 1 kHz), thereby enabling simultaneous measurement of mass spectra with and without laser light. Upon photon absorption, all adsorbed He atoms are typically lost from ${\text{Cs}}_{m}{\text{He}}_{n}^{+}$, and an optical photodissociation spectrum is derived from the depletion signal. A detailed description of the experiment can be found in the ESI† (Fig. S9) and elsewhere.^62^

To obtain a quantum chemical description of the optical spectra, ground state structures of ${\text{Cs}}_{m}{\text{He}}_{n}^{+}$ ions were first modelled at the coupled cluster singles doubles (CCSD) level with the def2QZVP basis set on Cs and def2TZVP on He. The position of the weakly bound He atoms, residing in shallow potential wells, is very sensitive to the quality of the basis set used on Cs. With the triple-zeta quality basis set def2TZVP used on Cs, a bent structure is predicted for Cs_2_He^+^; a linear Cs_2_He^+^ structure is obtained with the def2QZVP basis set on Cs and def2TZVP on He, and this does not change when the def2QZVPPD basis set is used on both Cs and He. To validate the theory as much as possible, excited states were modelled on various theory levels: time-dependent density functional theory (TDDFT) with the CAM-B3LYP functional; equation of motion – CCSD (EOM-CCSD); multi-reference configuration interaction (MRCI) with an active space of one electron in 5 or 17 orbitals, MRCI(1,5) and MRCI(1,17), respectively; def2QZVPPD basis set was used on all atoms for excited state calculations. Spin–orbit coupling was calculated using the state-interacting method as implemented in the Molpro program^63,64^ along with the ECP46MDF basis set.^65^ The *D*_2h_ symmetry group was used for calculations; irreducible representations (IRs) in the *D*_∞h_ symmetry group were reconstructed from the respective IRs in *D*_2h_ *D*_2h_. “Very Tight” optimization criteria were used for optimization; for optimization in the floppy excited state in Cs_2_He^+^, even stricter criteria for solving the EOM-CCSD equations were used, with convergence criteria of 10^−9^, 10^−11^ and 10^−10^ for energy, wavefunction and CCSD and ground-state *Z*-vector iterations, respectively. In all calculations, only 17 electrons in Cs_2_^+^ were treated explicitly, with the rest described within an effective core pseudopotential (ECP).

Purely dissociative as well as bound excited states are encountered in Cs_2_^+^. The absorption spectra of the dissociative states were modeled using the linear reflection principle (LRP) within the harmonic approximation^66–68^ and by the standard reflection principle projecting the vibrational wavefunction (calculated for the Cs_2_^+^ potential on a grid) onto the excited state potential energy surface. The spectra of the bound states were calculated using the Franck–Condon approximation within the harmonic regime as implemented in the Gaussian software^69^ or directly by calculating the overlap of the ground state vibrational wavefunction in the electronic ground state with both ground and excited vibrational wavefunctions in the electronically excited state.

Gaussian 16 was used for TDDFT and (EOM-)CCSD calculations, Molpro for MRCI and spin–orbit calculations.

## Results and discussion

**Fig. 1:**
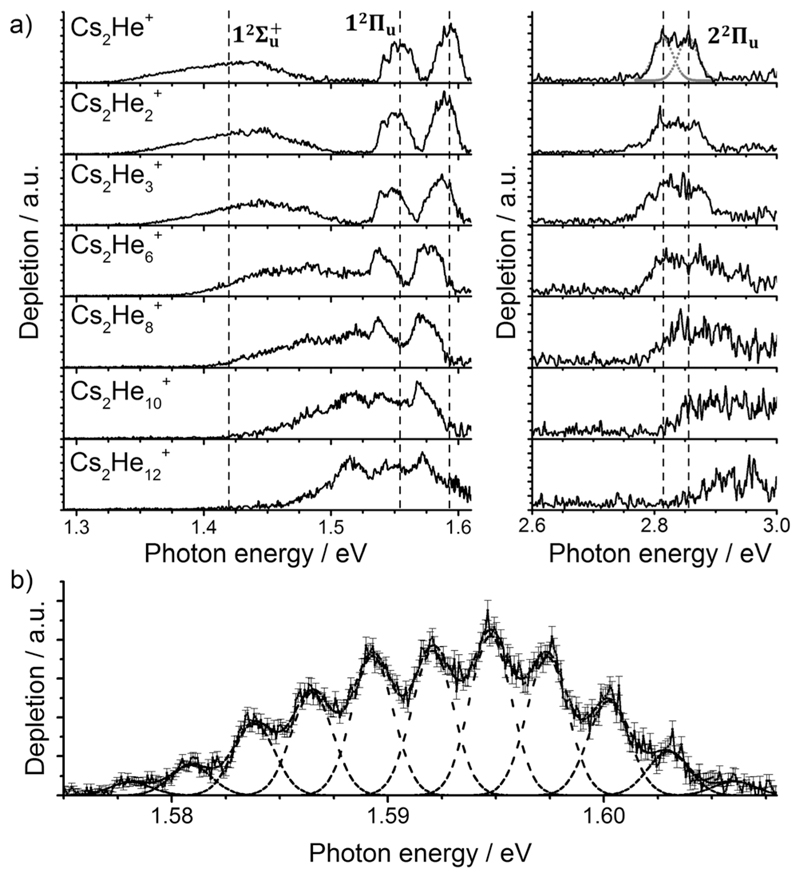
(a) Photodissociation spectra of ${\text{Cs}}_{2}{\text{He}}_{n}^{+}$ ions, *n* = 1, 2, 3, 6, 8, 10, 12 obtained as a differential spectrum with and without laser irradiation of clusters and scaled with laser power (see ESI† for details). A fit of the peak at 2.8 eV by two Gaussian functions is shown for *n* = 1. Band centers for Cs_2_He^+^ are marked by vertical lines to guide the eye. Electronic state assignment is based on calculations (Table 1). (b) Multiple Gaussian fit to the vibrational progression of the transition into the 1^2^Π_u_ state in Cs_2_He^+^ and determination of the vibrational spacing.

Let us start with the photodissociation spectrum of Cs_2_He^+^ (Fig. 1a). Upon electronic excitation, the He atom is lost, and the spectrum is recorded following the depletion of the ion signal. There are four clearly observed absorption bands at about 1.4, 1.55, 1.60 and 2.8 eV (Table 1). The band at 2.8 eV is further split, with spacing of about 40 meV (300 cm^−1^). The band at 1.60 eV has pronounced vibrational structure, with a spacing of 22 cm^−1^ (Fig. 1b); the broad bands at 1.4 and 2.8 eV are, on the other hand, structureless.

**Fig. 2:**
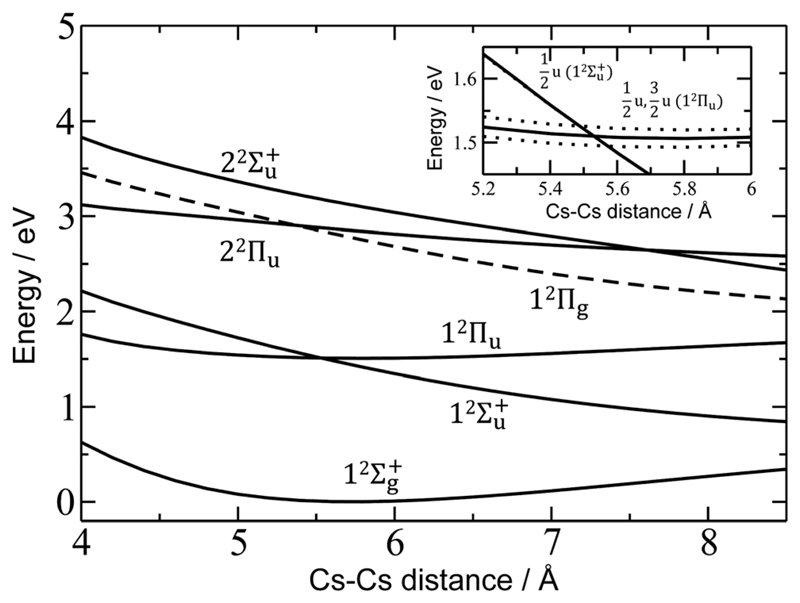
Potential energy curves in Cs_2_^+^ calculated at the EOM-CCSD/def2QZVPPD level. Excited states to which electronic transitions are allowed from the ground state are shown with full lines, forbidden ones with dashed lines. In the inset, the vicinity of the 1^2^Π_u_ minimum is shown, along with states including spin–orbit coupling (dotted lines, calculated at the MRCI(1,17)/ECP46MDF level of theory). Only selected states are shown. See the ESI† for excluded electronic states and method benchmarking.

For a more quantitative interpretation and reliable assignment of these features, we performed EOM-CCSD and MRCI calculations and analyzed the contribution of spin–orbit coupling effects. Fig. 2 shows the potential energy curves of Cs_2_^+^. The Cs_2_^+^ ion has *D*_∞h_ symmetry and, when disregarding spin–orbit coupling, its ground electronic state is $1^{2}{\text{Σ}}_{\text{g}}^{+}$. From this state, the only symmetry allowed transitions are perpendicular and parallel transitions into ^2^Π_u_ and ${\;}^{2}\Sigma_{\text{u}}^{+}$ states, respectively. Within 3 eV, two ${\;}^{2}\Sigma_{\text{u}}^{+}$ and two ^2^Π_u_ states can be found. Among these states, only the 1^2^Π_u_ state is bound, the others are dissociative. When accounting for spin–orbit effects (see Table S3 in the ESI†), the Σ states are only slightly affected, with shifts of up to 5 meV. However, the doubly-degenerate Π states split into two states, separated by ~10–35 meV (80–280 cm^−1^).

Comparison of the calculations with experiment, summarized in Table 1, allows us to assign the three main bands observed in the experiment as $1^{2}{\text{Σ}}_{\text{u}}^{+}$, 1^2^Π_u_, and 2^2^Π_u_, reproducing the excitation energy to within 0.1 eV. The fourth allowed transition into $2^{2}{\text{Σ}}_{\text{u}}^{+}$ at ~3.1 eV (see Table S2, ESI†) is not observed in the experiment due to insufficient laser power in this region. For the dissociative states $1^{2}{\text{Σ}}_{\text{u}}^{+}$ and 2^2^Π_u_, calculations within the reflection principle approximation also predict a realistic width of the observed bands, see Table 1.

As mentioned above, the 1^2^Π_u_ state is bound and, therefore, the measured photodissociation spectrum reflects its vibrational structure. The 1^2^Π_u_ state is calculated to have a very similar equilibriumdistance as the $1^{2}{\text{Σ}}_{\text{g}}^{+}$ ground state (Fig. 2). The excited state potential well is less steep, reflected in the calculated harmonic vibrational frequencies of 30 and 20 cm^−1^ for the $1^{2}{\text{Σ}}_{\text{g}}^{+}$ and 1^2^Π_u_ state, respectively, for Cs_2_^+^ (at the (EOM-)CCSD/def2QZVPPD level). The respective values for Cs_2_^+^He are 31 and 19 cm^−1^, with a stronger Cs_2_^+^–He interaction in the excited states, shortening the Cs–He distance from 4.4 Å in the $1^{2}{\text{Σ}}_{\text{g}}^{+}$ state to 4.0 Å in 1^2^Π_u_. The harmonic Cs_2_^+^…He vibration is located at 30 cm^−1^. The calculated frequency in the 1^2^Π_u_ state of 19 cm^−1^ fits well to the measured spacing of 22 cm^−1^. Note that only the higher 3/2u(1^2^Π_u_) state shows clear vibrational resolution shown in Fig. 1b. The 1/2u(1^2^Π_u_) state interacts with the repulsive 1/2u($1^{2}{\text{Σ}}_{\text{u}}^{+}$) state, and the potential energy surface will be deformed due to the avoided crossing of the states. From a computational perspective, the equilibrium distance in the 1^2^Π_u_ state depends heavily on the choice of the basis set (Fig. S4, ESI†). As a direct consequence, our attempts to reproduce the experimental spectrum shown in Fig. 1b using Franck–Condon simulations failed to grasp correctly the relative intensity of the contributing peaks (see the ESI† for details).

**Fig. 3:**
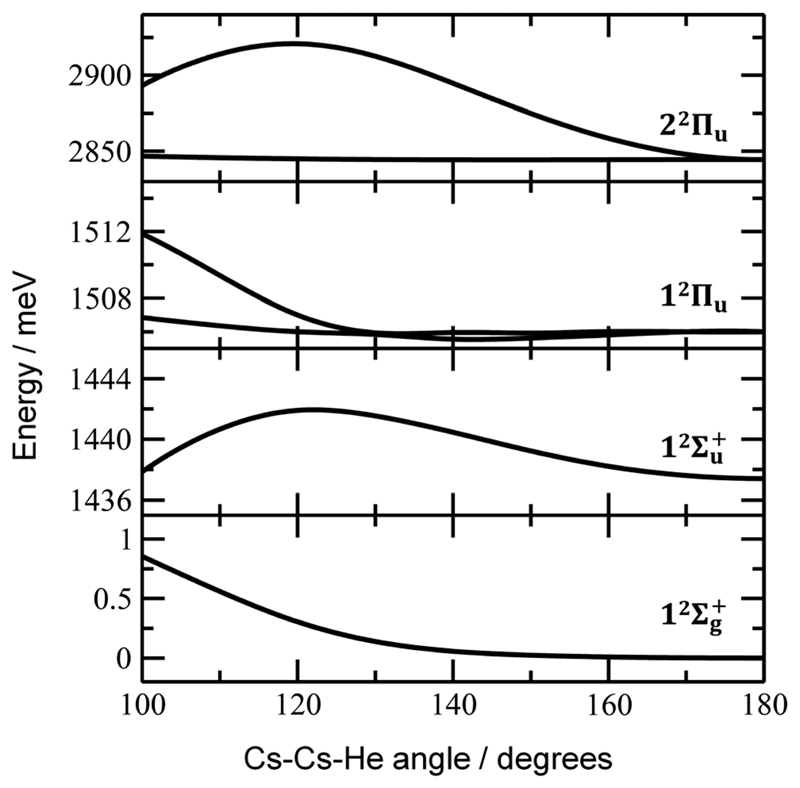
Dependence of ground state and excited state energies in Cs_2_He^+^ on the Cs–Cs–He angle. Calculated at the EOM-CCSD/def2QZVPPD level of theory with ground-state structures optimized at the CCSD/def2QZVP(Cs),def2TZVP(He) level with a constrained Cs–Cs–He angle. Spin–orbit effects were neglected.

**Fig. 4:**
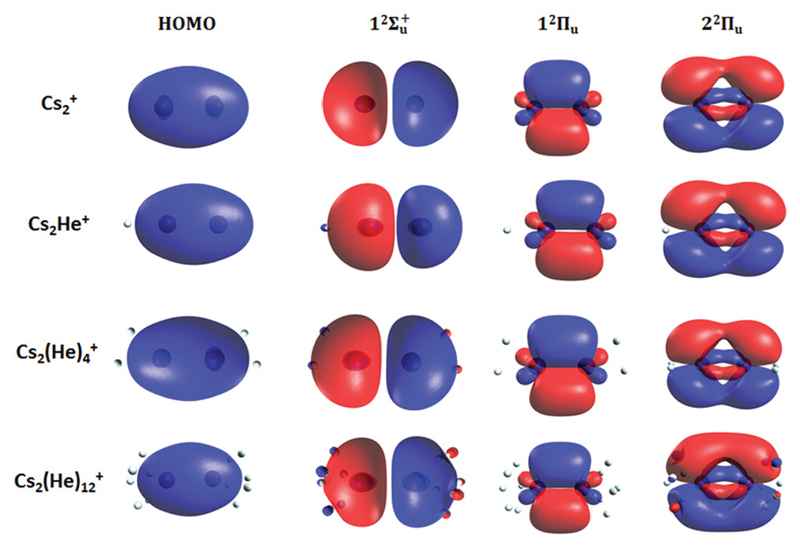
Highest occupied molecular orbital (HOMO) and natural transition orbitals for ${\text{Cs}}_{2}{\text{He}}_{n}^{+}$ ions calculated at the CAM-B3LYP/def2QZVPPD level of theory. A low iso value (0.005) was chosen so that orbital deformation induced by helium is clearly visible.

Finally, the calculated weak spin–orbit splitting of the ^2^Π_u_ states results into two states 1/2u and 3/2u with a different Ω value following Hund’s rule 3. The splitting is well reproduced for 1^2^Π_u_ (see also inset in Fig. 2). For 2^2^Π_u_, the calculated spin– orbit splitting is significantly smaller than the experimentally observed splitting. These observations can be rationalized if one takes into account the additional splitting induced by the asymmetric distribution of the He atom relative to the singly occupied molecular orbital (SOMO) in the excited state. Fig. 3 illustrates how the energy of the several electronic states in Cs_2_He^+^ depends on the position of the He atom in Cs_2_He^+^. For this calculation, the Cs–Cs–He angle is fixed, and the Cs–Cs and Cs–He distances are optimized in the electronic ground state, $1^{2}{\text{Σ}}_{\text{g}}^{+}$. From 180° to 150°, the ground state energy hardly changes, *i.e*. the He atom moves more or less freely in this range of Cs–Cs–He angles. The repulsive $1^{2}{\text{Σ}}_{\text{u}}^{+}$ state reacts more sensitively to the position of the He atom, and this effect contributes to the broadening of the absorption band, which is 19 meV larger than expected from the linear reflection principle approximation, Table 1. For the 1^2^Π_u_ and 2^2^Π_u_ states, the degeneracy of the two contributing π orbitals (see Fig. 4) is lifted by the presence of the He atom, which leads to an additional splitting. For 1^2^Π_u_, this effect is negligible down to 150° Cs–Cs–He angle, because the He atom does occupy the same space as the singly occupied π orbitals. In contrast, the SOMO of the 2^2^Π_u_ state is much more diffuse, and in one of the two orientations exhibits significant overlap with the position of the He atom. The splitting of the two orientations of the SOMO in the 2^2^Π_u_ state amounts to 31 meV at 150°, which constitutes the dominant contribution to the experimentally observed splitting. For larger clusters, the same effect is responsible for the significant broadening of the 2^2^Π_u_ band.

Let us now concentrate on the shift of the spectra induced by solvation, *i.e*. the He matrix shift. As shown in Fig. 1a and Table 2, pronounced shifts to higher energies are recorded for the $1^{2}{\text{Σ}}_{\text{u}}^{+}$ and 2^2^Π_u_ states, reaching values of more than 0.1 eV already for Cs_2_He_10_^+^. Note also that the shift in the $1^{2}{\text{Σ}}_{\text{u}}^{+}$ state exceeds the He binding energy. In the *n* = 1–10 range, the energy of the $1^{2}{\text{Σ}}_{\text{u}}^{+}$ state shifts linearly with solvation by 12 meV (100 cm^−1^) per helium atom. The 2^2^Π_u_ band at ~2.8 eV seems to shift slightly non-linearly, its structure is however not well resolved in the experiment for larger ions. The 1^2^Π_u_ state at 1.6 eV, on the other hand, is shifted only by ~20 meV (160 cm^−1^) to lower energies within *n* = 1–12 (Table 2).

The shifts can be directly connected to the interplay between the position of He atoms around the Cs_2_^+^ ion and the character of the respective excited state. Our calculations show that Cs_2_^+^ is preferentially solvated by He atoms on its ends, along the Cs–Cs axis for the smallest ions (see Fig. 4). For Cs_2_He^+^ and Cs_2_He_2_^+^, the most stable configuration is linear, but deformation from linearity down to a Cs–Cs–He angle of 150° requires very little energy, see Fig. 3; in Cs_2_He_12_^+^, conformations with six and five/seven He atoms on each end are the most stable ones (Table S1, ESI†). As expected, the binding energy of helium atoms is low, within 2.4–3.1 meV He^−1^ for up to 12 helium atoms. In addition to the shifts, the presence of a large variety of almost isoenergetic isomers leads to significant broadening of the spectra upon solvation (see Fig. S1, ESI†).

Due to Pauli repulsion, the presence of He atoms deforms the orbitals of both ground and excited electronic states that extend along the Cs–Cs axis, shifting them to higher energy. This is the case for the HOMO orbital as well as the orbitals corresponding to excitations into the $1^{2}{\text{Σ}}_{\text{u}}^{+}$ and 2^2^Π_u_ states (Fig. 4). The diffuse orbitals in the excited state are deformed more than the HOMO orbital, shifting the excitation energy considerably to higher values (Fig. 1a). The π orbital corresponding to the excitation into the 1^2^Π_u_ state at 1.6 eV represents a different case. Here, the surrounding helium atoms do not approach the orbital and the shift to lower energies reflects the slight destabilization of the HOMO orbital. Our calculations can reproduce the shifts for excitations into $1^{2}{\text{Σ}}_{\text{u}}^{+}$ and under-estimate those observed for 1^2^Π_u_ (Table 2); for *n* = 12, the sudden increase observed in the 1^2^Π_u_ experimental shift might be induced by the fitting procedure (see Fig. S12, ESI†) or appearance of new isomers. This is most likely caused by the neglect of He delocalization around the Cs_2_^+^ core due to zero-point motion and the presence of multiple isomers.

## Conclusions

In contrast to photodissociation of bare Cs_2_^+^, helium tagging gives experimental access to the 3/2u component of the 1^2^Π_*u*_ state, which features a pronounced vibrational progression with a Cs–Cs frequency of 22 cm^−1^ in the excited state. According to quantum chemical calculations, He considerably changes the spectrum already for the Cs_2_He^+^ ion, inducing splitting of bands due to both solvation and dynamic effects. When more helium atoms are added, shifts exceeding 0.1 eV, *i.e*. almost 1000 cm^−1^, are induced, and the 2^2^Π_*u*_ significantly broadens. Themagnitude and even direction of the shift are extremely sensitive to both the nature of the electronic transition as well as the position of the solvating atoms. Our calculations show that the shifts are particularly pronounced if He occupies a position in the electronic ground state that carries large electron density in the excited state. Despite its extremely weak interaction, He is capable of inducing significant matrix shifts in electronic excitation spectra, due to Pauli repulsion, as already pointed out before.^70,71^ This effect is used to derive information both on the shape of excited state molecular orbitals as well as solvation patterns.

## Supplementary Material

† Electronic supplementary information (ESI) available: Relative stabilities of calculated isomers, method benchmarks, spin–orbit couplings, complete experimental spectra, Cartesian coordinates. See DOI: 10.1039/c9cp04790e

Supporting Information

## Figures and Tables

**Table 1:** Experimental and theoretical properties of excited states in Cs_2_^+^ and Cs_2_He^+^: energy position *E* (in eV), width *w* of the Gaussian peak fit (= 2*σ*), experimental splitting Δ*E*_split_, spin–orbit splitting Δ*E*_SO_ (all in meV). Excitation energies were calculated at the EOM-CCSD/def2QZVPPD level of theory, spectral width was modelled within reflection principle approximation, spin–orbit coupling at the MRCI(1,17)/ECP46MDF level

State		1^2^ $\sum_{\text{u}}^{+}$	1^2^Π_u_	2^2^Π_u_
Experiment, Cs_2_He^+^	*E*	1.41	1.55; 1.59	2.82; 2.86
	*w*	83	–	39; 30
	Δ*E*_split_	–	39	42
Theory, Cs_2_^+^	*E*	1.43	1.51	2.85
	*w*	64	–	26
	Δ*E*_so_	–	28	13
Theory, Cs_2_He^+^	*E*	1.44	1.51	2.84
	Δ*E*_so_	–	28	14

**Table 2:** Experimental and calculated energy shift of excited states upon solvation (in meV) with respect to Cs_2_He^+^. See Fig. S12 for experimental fits. Electronic states are correlated to 1^2^ $\sum_{\text{u}}^{+}$ and 1^2^Π_u_ states in Cs_2_^+^. Vertical transitions were calculated at the EOM-CCSD/def2QZVPPD//CCSD/def2QZVP(Cs), def2TZVP(He) level of theory. For the 1^2^Π_u_ state, the average calculated value of the two SOMO orientations is given. Isomers with equal distribution of He atoms to each Cs_2_^+^ end are chosen for the calculation

*n*_He_	Experiment		Theory	
	1^2^ $\sum_{\text{u}}^{+}$	1^2^Π_u_	1^2^ $\sum_{\text{u}}^{+}$	1^2^Π_u_
2	11	–3.4; –3.7	6	–0.8
3	23	–6.1; –6.6	15	–2.1
6	59	–13.3; –15.2	41	–6.1
10	98	–9.2; –19.3	96	–12.0
12	124	–18.5; –41.0	109	–14.2
