# Supplementary material for: Considerable matrix shift in the electronic transitions of helium-solvated cesium dimer cation Cs2Hen+ †
Source: Phys Chem Chem Phys. Author manuscript; Available in PMC 2020 Nov 8. (PMC7116336; doi:10.1039/c9cp04790e)
Supplement: Supporting Information [file EMS102275-supplement-Supporting_Information.pdf]

Electronic Supplementary Material (ESI) for Physical Chemistry Chemical Physics.
This journal is © the Owner Societies 2019

Supporting Information

Considerable Matrix Shift in the Electronic Transitions of Helium-Solvated Cesium Dimer Cation $Cs_2He_n^+$

Lorenz Kranabetter,[a] Nina K. Bersenkowitsch,[a] Paul Martini,[a] Michael Gatchell,[b] Martin Kuhn,[a] Felix Laimer,[a] Arne Schiller,[a] Martin K. Beyer,[a] Milan Ončák,[a*] Paul Scheier[a*]

a – Institut für Ionenphysik und Angewandte Physik, Universität Innsbruck, Technikerstr. 25, A-6020 Innsbruck

b – Department of Physics, Stockholm University, 106 91 Stockholm, Sweden

E-Mail: Milan.Oncak@uibk.ac.at, Paul.Scheier@uibk.ac.at

Contents

1. Isomers of $Cs_2He_n^+$ and shift of excited states with solvation 2
2. Computational treatment of excited states 7
3. Spin-orbit coupling in Cs_2^+ 10
4. Modeling of absorption spectra 11
5. Experimental details and complete experimental spectra 12
6. Cartesian coordinates of $Cs_2^+He_n$ clusters optimized at the CCSD/def2-QZVP(Cs),def2-TZVP(He) level of theory unless stated otherwise (in Ångstrom, energy in Hartree) 15

1. Isomers of $Cs_2He_n^+$ and shift of excited states with solvation

Figure S1 includes structures of $Cs_2He_n^+$, n = 1–12, clusters optimized at the CCSD/def2TZVP level of theory. For each n, all distributions of helium atoms on both ends of the Cs-Cs axis were considered. Table S1 shows the relative stability of the isomers as well as excitation energy into four allowed electronic states.

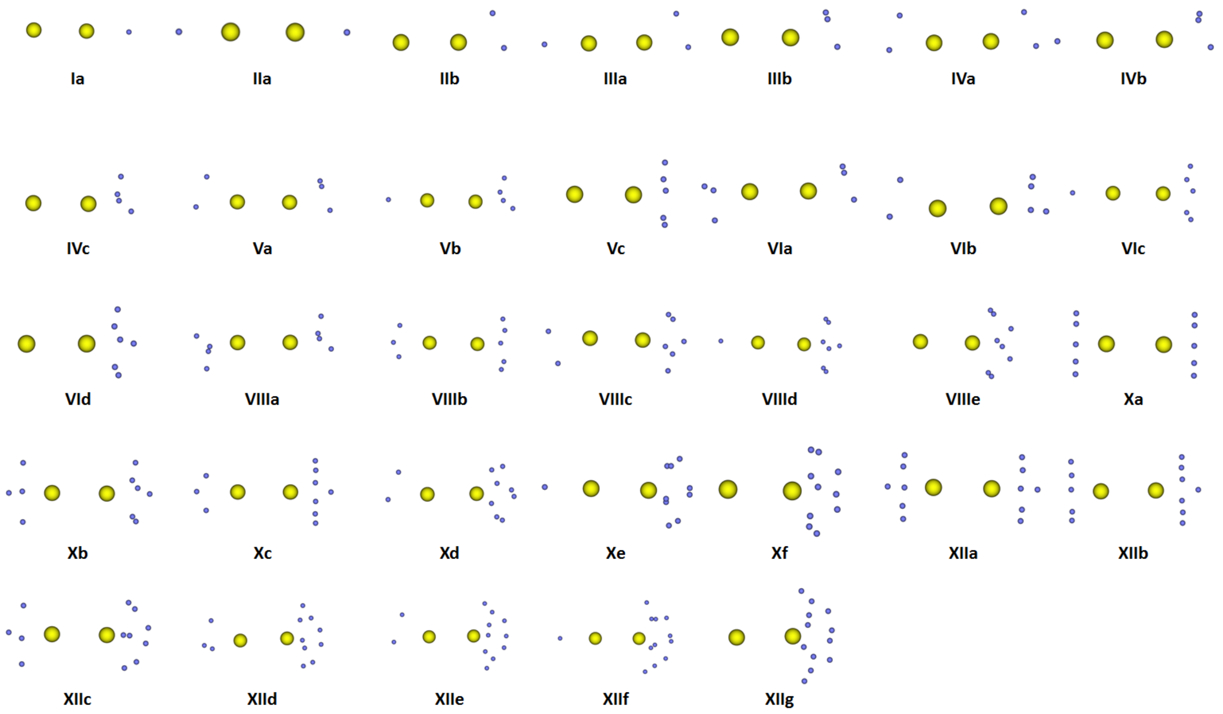

Figure S1 – Selected structures of $Cs_2He_n^+$ optimized at the CCSD/def2QZVP(Cs),def2TZVP(He) level of theory.

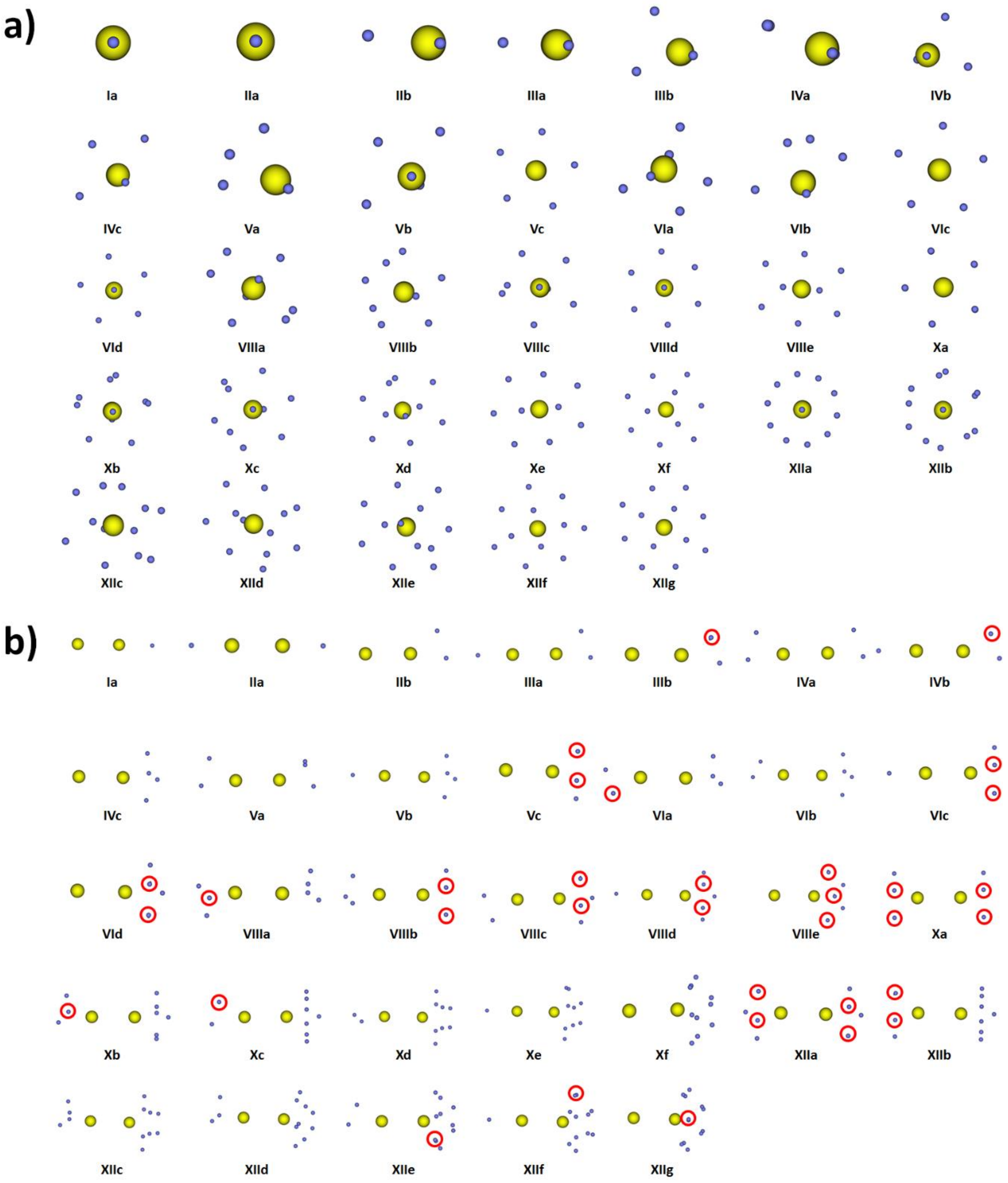

Figure S2 – a) Structures shown in Figure S1 visualized in projection along the Cs-Cs axis. b) Projections of structures shown in Figure S1 into a Cs-Cs-He plane. Red circles show two helium atoms at the same position.

Table S1 – Relative energies of isomers ΔE, average solvation energy per He atom $E_{\mathrm{solv,aver}}$ and excitation energies E_{exc} of $Cs_2He_n^+$ isomers shown in Figure S1. Relative stability and excitation energies were calculated at the CCSD/def2QZVP(Cs),def2TZVP(He) and EOM-CCSD/def2QZVPPD levels, respectively. Excited states are correlated to the electronic states in Cs_2^+. Spin orbit coupling was neglected.

isomer	ΔE [meV]	$E_{\mathrm{solv,aver}}$ [meV]	E_{exc} [eV] $1^2\Sigma_u^+$	$1^2\Pi_u$		$2^2\Pi_u$		$2^2\Sigma_u^+$
Cs_2^+	–	–	1.43	1.51		2.85		3.11
**Ia**	–	-3.07	1.44	1.51		2.84		3.22
**IIa**	0.00	-3.07	1.44	1.51		2.84		3.32
**IIb**	0.35	-2.89	1.45	1.50	1.51	2.84	2.90	3.28
**IIIa**	0.00	-2.94	1.45	1.50	1.50	2.84	2.91	3.36
**IIIb**	0.74	-2.70	1.45	1.50	1.50	2.88	2.90	3.33
**IVa**	0.00	-2.87	1.46	1.50	1.50	2.83	2.98	3.39
**IVb**	0.34	-2.78	1.46	1.50	1.50	2.85	2.88	3.40
**IVc**	1.19	-2.57	1.46	1.50	1.50	2.90	2.96	3.36
**Va**	0.00	-2.77	1.47	1.50	1.50	2.86	2.88	3.42
**Vb**	0.51	-2.66	1.47	1.50	1.50	2.86	2.96	3.42
**Vc**	1.00	-2.57	1.48	1.50	1.50	3.00	3.00	3.30
**VIa**	0.00	-2.70	1.48	1.50	1.50	2.90	2.91	3.47
**VIb**	0.16	-2.67	1.48	1.50	1.50	2.86	2.95	3.43
**VIc**	0.29	-2.65	1.48	1.50	1.50	3.00	3.00	3.37
**VId**	1.68	-2.42	1.48	1.50	1.50	3.02	3.02	3.41
**VIIIa**	0.38	-2.57	1.50	1.50	1.50	2.91	2.99	3.55
**VIIIb**	0.00	-2.61	1.50	1.50	1.50	3.01	3.05	3.44
**VIIIc**	0.71	-2.52	1.50	1.50	1.50	3.01	3.03	3.51
**VIIId**	1.63	-2.41	1.50	1.50	1.50	3.06	3.06	3.46
**VIIIe**	3.83	-2.13	1.50	1.50	1.50	2.78	2.79	3.39
**Xa**	0.00	-2.56	1.53	1.49	1.49	3.07	3.07	3.38
**Xb**	0.88	-2.47	1.52	1.49	1.49	3.01	3.04	3.53
**Xc**	1.33	-2.43	1.52	1.49	1.50	3.06	3.10	3.52
**Xd**	2.81	-2.28	1.52	1.50	1.50	3.07	3.14	3.50
**Xe**	4.08	-2.15	1.51	1.50	1.50	3.08	3.19	3.47
**Xf**	6.15	-1.94	1.51	1.50	1.50	3.18	3.20	3.39
**XIIa**	0.18	-2.40	1.55	1.49	1.49	3.08	3.08	3.58
**XIIb**	0.00	-2.42	1.55	1.49	1.49	3.11	3.11	3.45
**XIIc**	1.68	-2.28	1.54	1.49	1.50	3.10	3.16	3.53
**XIId**	2.46	-2.21	1.53	1.49	1.50	3.10	3.20	3.53
**XIIe**	3.81	-2.10	1.53	1.50	1.50	3.18	3.20	3.49
**XIIf**	6.17	-1.90	1.52	1.50	1.50	3.20	3.24	3.47
**XIIg**	8.01	-1.75	1.51	1.50	1.50	3.24	3.25	3.39

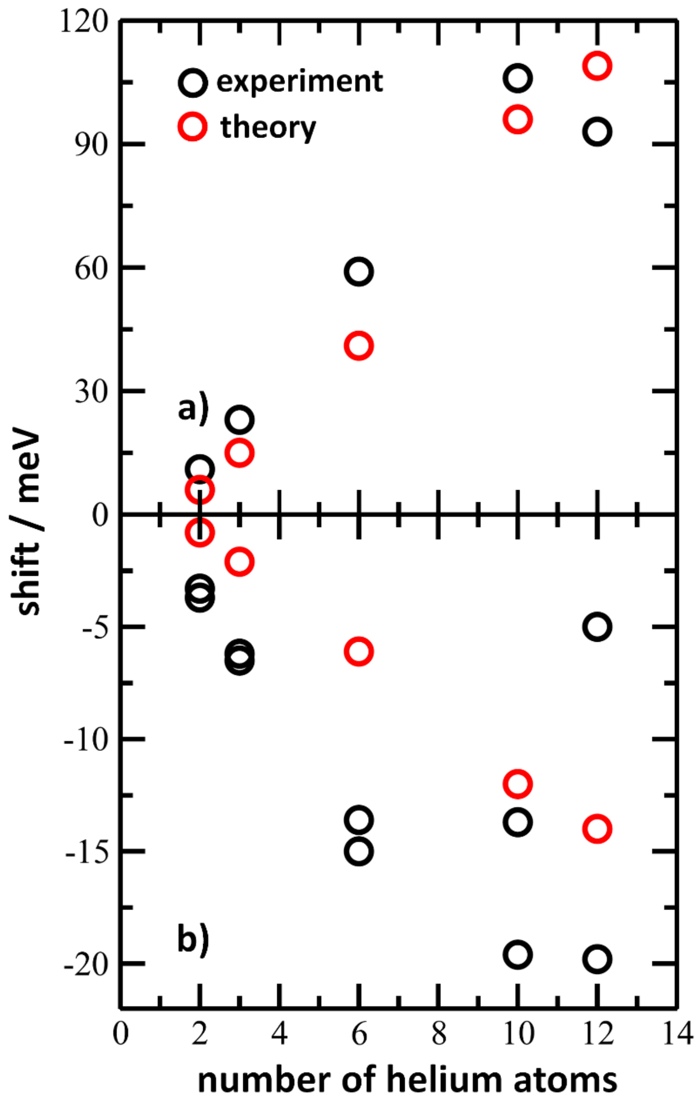

Figure S3 – Plotted shifts shown in Table 2 for a) $1^2\Sigma_u^+$ and b) $1^2\Pi_u$ states.

Table S2 – Correlation of the most important electronic states between Cs_2^+ and Cs_2He^+. Calculated at the EOM-CCSD/def2QZVPPD//CCSD/def2QZVP(Cs),def2TZVP(He) level of theory. Energy *E* (in eV) and oscillator strength *f* are given.

Cs_2^+			Cs_2He^+		
state	*E*	*f*	state	*E*	*f*
$1^2\Sigma_u^+$	1.432	0.341	$2^2\Sigma^+$	1.437	0.339
$1^2\Pi_u$	1.507	0.280	$1^2\Pi$	1.506	0.280
$2^2\Pi_u$	2.848	0.038	$3^2\Pi$	2.845	0.037
$1^2\Sigma_u^+$	3.112	0.033	$5^2\Sigma^+$	3.221	0.036

Table S3 – Cs-He bond length (in Å) for selected isomers optimized with the CCSD and CCSD(T) method along with the def2QZVP(Cs),def2TZVP(He) basis set. It can be seen that the Cs-He interaction is not considerably affected by the inclusion of triplets.

isomers	CCSD	CCSD(T)
**Ia**	4.6090	4.6083
**IIa**	4.6115	4.6108
**IIb**	4.4742; 4.5803	4.4734; 4.5795
**IIIa**	4.4765; 4.5832; 4.6144	4.4753; 4.5822; 4.6136
**IIIb**	4.4422 (2x); 4.5408	4.4408; 4.4411; 4.5398

2. Computational treatment of excited states

In Figure S4, we compare two methods (MRCI, EOM-CCSD) and two basis sets (def2TZVP and def2QZPPD) for calculation of the excited states in Cs_2^+. Due to the loosely bound $1^2\Pi_u$ excited state, the choice of the basis set represents an issue. In Figure S4a,b, we show that the position of the $1^2\Pi_u$ minimum changes considerably when passing from the def2TZVP to the def2QZVPPD basis set. On the other hand, both methods give similar results when using the def2QZVPPD basis set, with the same shape of the potential energy curves; however, the EOM-CCSD method predicts slightly higher excitation energies. This might be traced to the limited active space size as well as inclusion of 17 electronic states within the state averaging scheme. Figures S3, S4 compare potential energy surface of all 17 states for EOM-CCSD and MRCI.

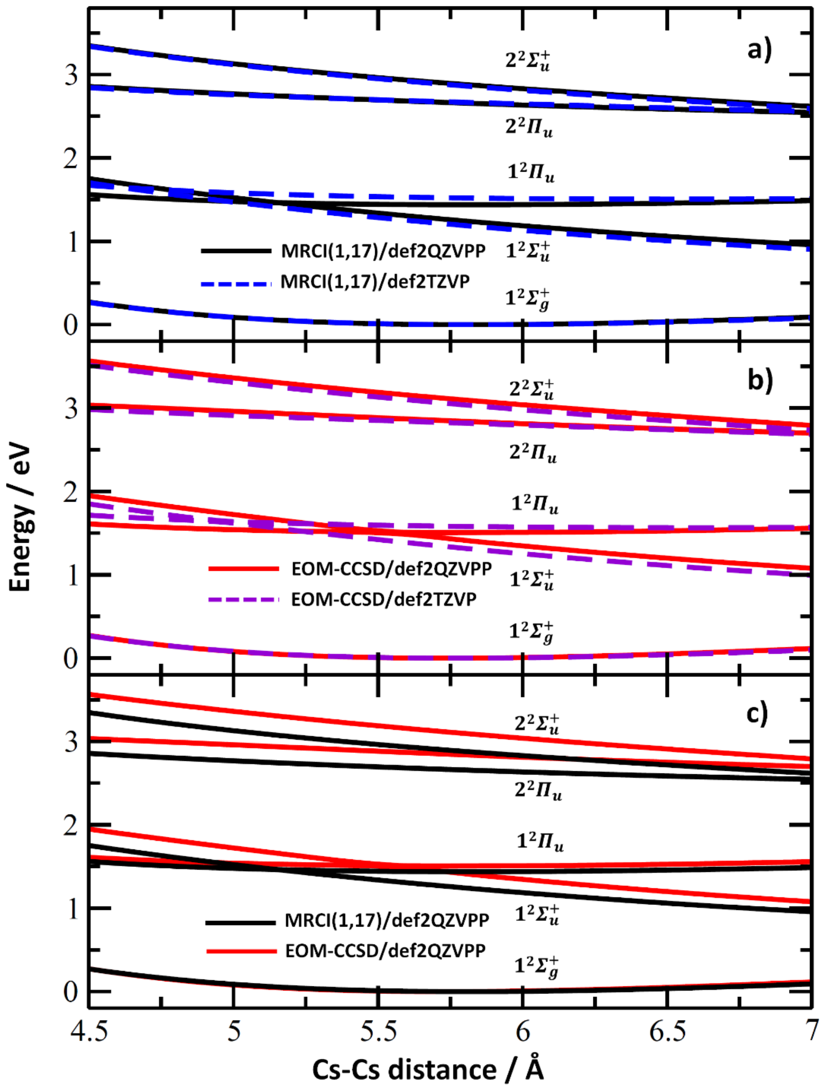

Figure S4 – Potential energy curves for Cs_2^+ employing different methods and basis sets.

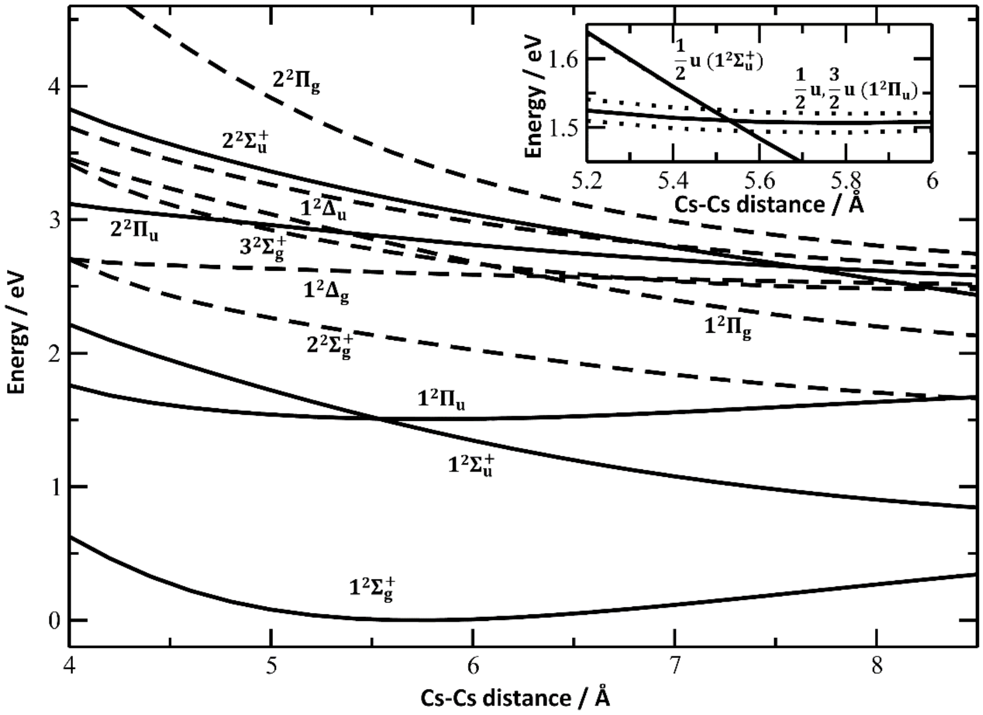

Figure S5 – Potential energy curves in Cs_2^+ calculated at the EOM-CCSD/def2QZVPPD level. Allowed transitions are shown with full lines, forbidden ones with dashed lines. In the inset, the vicinity of the $1^2\Pi_u$ minimum is shown, along with states including spin-orbit coupling (dotted lines, calculated at the MRCI(1,17)/ECP46MDF level of theory).

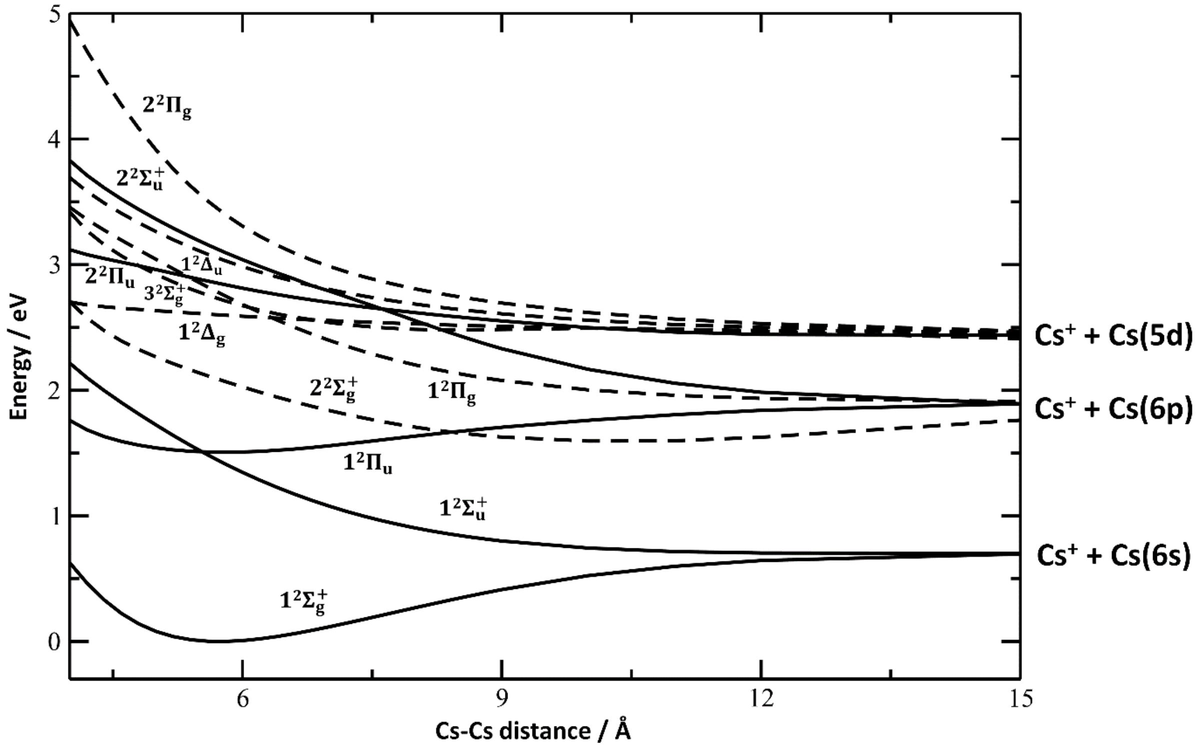

Figure S6 – Potential energy curves in Cs_2^+ calculated with dissociation asymptotes. See Figure S5 for computational details.

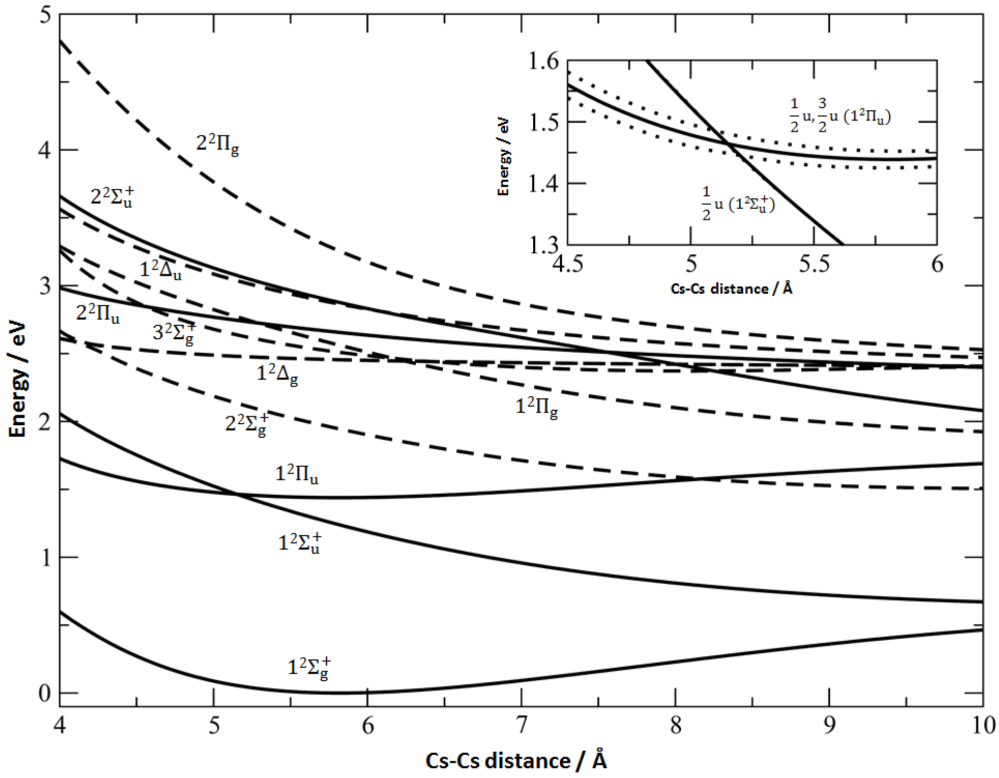

Figure S7 – Potential energy curves in Cs_2^+ calculated at the MRCI(1,17)/def2QZVPPD level. Allowed transitions are shown with full lines, forbidden ones with dashed lines. In the inset, the vicinity of the $1^2\Pi_u$ minimum is shown, along with states including spin-orbit coupling (dotted lines, calculated at the MRCI(1,17)/ECP46MDF level of theory).

3. Spin-orbit coupling in Cs_2^+

Table S4 – Electronic states in Cs_2^+ with and without spin-orbit coupling along with the spin-orbit energy shift in meV. Calculated for the Cs-Cs interatomic distance of 5.7304 Å at the MRCI(1,17)/ECP46MDF level.

original state	SO state	Δ*E*
$1^2\Sigma_g^+$	$\frac{1}{2}g$	-0.17
$1^2\Sigma_u^+$	$\frac{1}{2}u$	-0.05
$1^2\Pi_u$	$\frac{1}{2}u$	-13.95
$1^2\Pi_u$	$\frac{3}{2}u$	13.81
$2^2\Sigma_g^+$	$\frac{1}{2}g$	-1.19
$1^2\Delta_g$	$\frac{3}{2}g$	-4.72
$1^2\Delta_g$	$\frac{5}{2}g$	4.65
$3^2\Sigma_g^+$	$\frac{1}{2}g$	-4.42
$1^2\Pi_g$	$\frac{1}{2}g$	-14.27
$1^2\Pi_g$	$\frac{3}{2}g$	19.69
$2^2\Pi_u$	$\frac{1}{2}u$	-7.17
$2^2\Pi_u$	$\frac{3}{2}u$	6.21
$1^2\Delta_u$	$\frac{3}{2}u$	-6.86
$1^2\Delta_u$	$\frac{5}{2}u$	7.02
$2^2\Sigma_u^+$	$\frac{1}{2}u$	0.98
$2^2\Pi_g$	$\frac{1}{2}g$	-7.42
$2^2\Pi_g$	$\frac{3}{2}g$	7.83

4. Modeling of absorption spectra

To describe purely dissociative states ($1^2\Sigma_u^+$, $2^2\Sigma_u^+$, $2^2\Pi_u$), we use the reflection principle (Figure S8a). Comparing the results of the linearized reflection principle with the ones obtained through direct solution of the Schrödinger equation on a grid, we can see that even the approximate treatment provides absorption spectra of good quality. For the bound $1^2\Pi_u$ electronic state, a quantitative description of the experimentally measured absorption spectrum within Franck-Condon simulation is more complicated. The position of the excited state minimum depends sensitively on the basis set quality and method employed (Figure S4), influencing Franck-Condon integrals. For these reasons, quantitative agreement with the measured spectra could not be reached (Figure S8b).

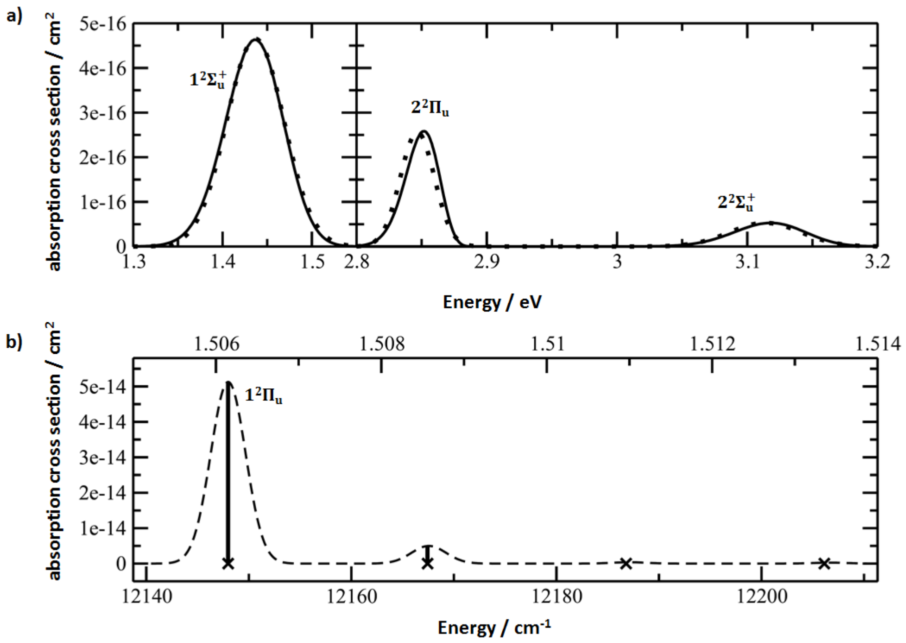

Figure S8 – a) Absorption spectra of $1^2\Sigma_u^+$, $2^2\Sigma_u^+$, $2^2\Pi_u$ states in Cs_2^+ based on the reflection principle approximation. In full lines, calculation with direct solution of the Schrödinger equation in the ground state and using the full potential energy surface of the excited states is shown. Dotted lines show the results with the linearized reflection principle. Calculated at the (EOM-)CCSD/def2QZVPPD level of theory. Spin-orbit coupling was neglected. b) Franck-Condon simulations for transition into the $1^2\Pi_u$ state in Cs_2^+. Calculation using the harmonic approximation with half width at half maximum of 2 cm^{-1} is shown in dashed lines. Bars show calculations based on full potential energy surface, the intensity of the second power of Franck-Condon integrals is scaled to the intensity calculated using the harmonic approximation; the excitation energy is also shifted to match the onset of the spectrum in the harmonic approximation. Crosses show the respective transition positions. Calculated at the (EOM-)CCSD/def2QZVPPD level. Spin-orbit coupling was neglected.

5. Experimental details and complete experimental spectra

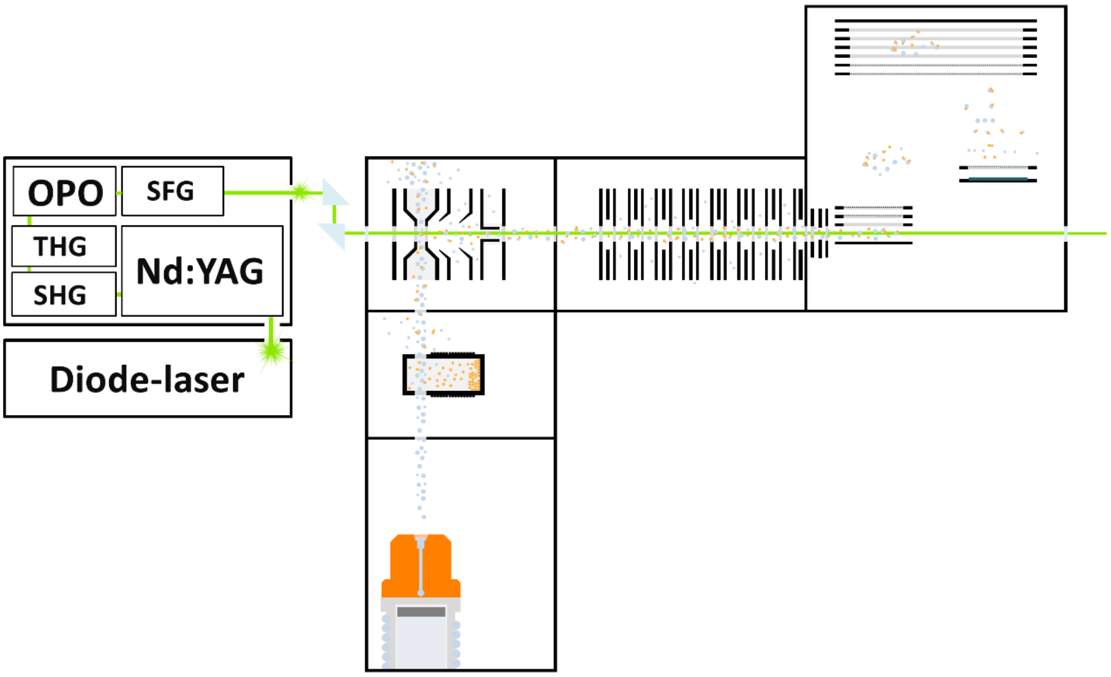

Figure S9: Scheme of the experimental setup.

The experimental setup utilizes helium nanodroplets to pick up Cs atoms and agglomerate them into small clusters. The droplets are produced via supersonic expansion of pre-cooled helium (27 bar, 9.95 K) through a 5 µm nozzle. After the helium nanodroplet beam is skimmed, it traverses a residually heated oven which evaporates the sample to the gas phase where it is picked up. The doped droplets are ionized via electron bombardment well after the cesium clusters have formed on their surface. The ionization dynamics yields the final helium-cesium complexes after the excess energy from the pickup and the ionization evaporates some helium (see *Phys. Rep.* 751 (2018) 1–90; *Phys. Chem. Chem. Phys.* 9 (2007) 4748–4770). The orthogonal extraction of the ions from the neutral beam facilitates merging of the laser light with the He tagged ions. The ion beam is then focused by an Einzel-lens-stack to be subsequently extracted into a time of flight mass spectrometer. The pulsed light source is operated in such a way that every 10th extraction into the time of flight apparatus is irradiated when passing the Einzel-lens-stack. The extractions of the time of flight are organized in blocks of 10 segments, where each segment is allocated into its own memory buffer. One of those buffers will collect the irradiated data while the other channels sequentially collect the reference spectra with a sampling rate of 1kHz. A differential spectrum is calculated from the recorded data and wavelength dependent mass traces are extracted to illustrate the wavelength dependent depletion of a single mass. Several filter and normalizations are applied to the raw data in order to account for laser power fluctuation and other effects. The laser light source is able to produce laser light of high brilliance from 210 to 2600 nm but the pulse power depends strongly on the wavelength. At certain wavelengths, the combination of absorption cross-section and laser-power does not allow conclusive statements on the existence of absorption lines.

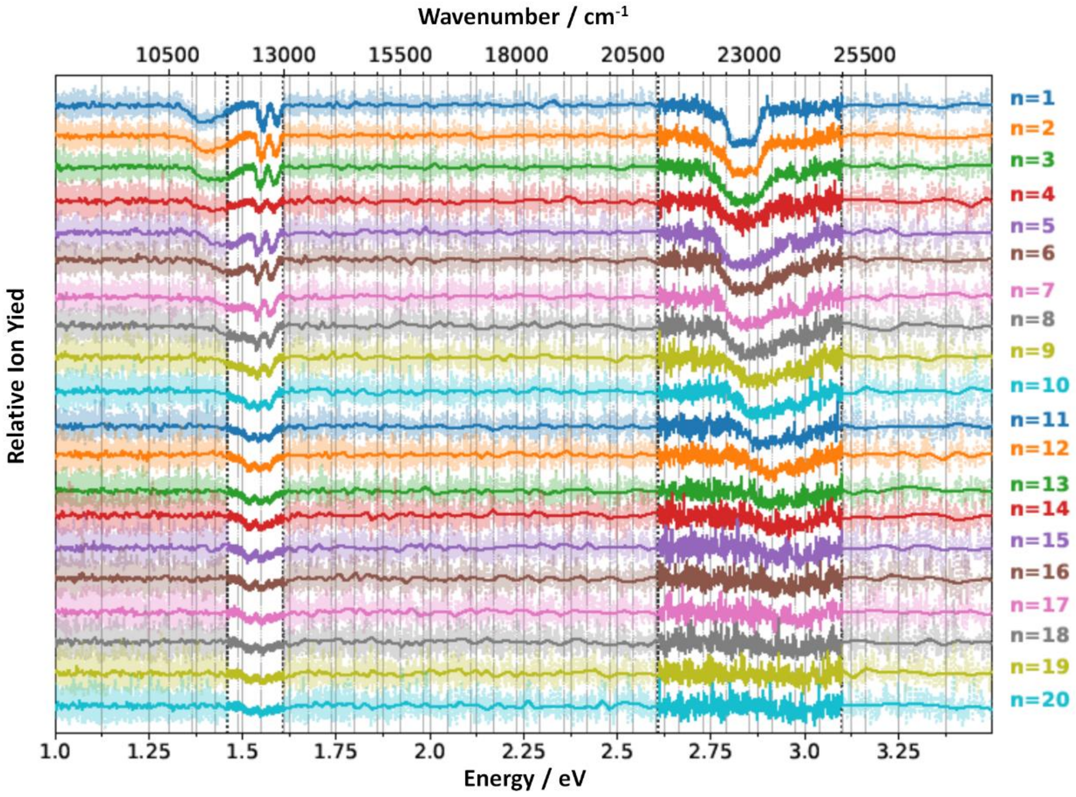

Figure S10: A survey of the raw depletion in the accessible energy interval reflecting the measurements with the highest available fidelity. Dashed lines indicate the border of the intervals of single measurements. The individual raw data was normalized to have a common background noise level and high noise measurements where smoothed (the transparent line indicates data without smoothing). This figure shows depletion which is not corrected for laser power.

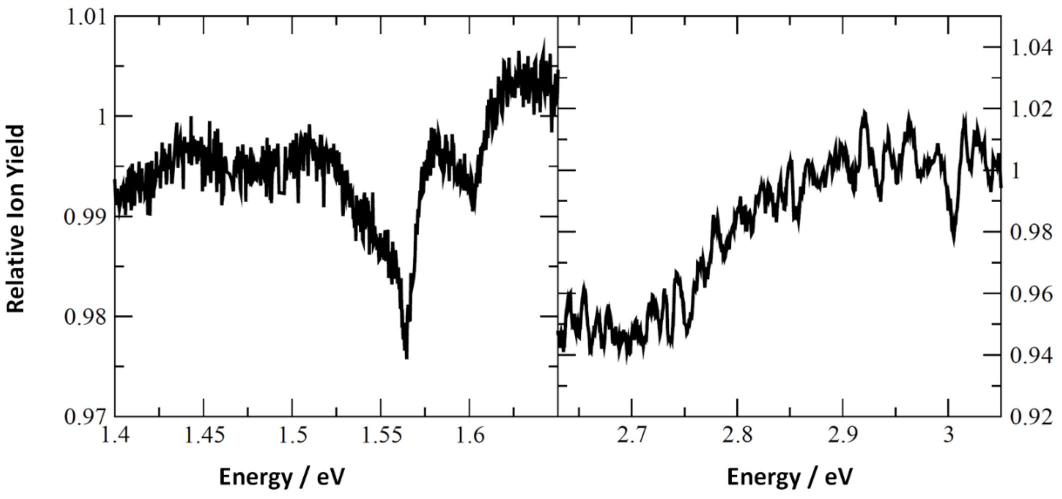

Figure S11: Ion depletion spectrum of Cs_2^+. This channel contains contributions from both the fragmentation of $Cs_2^+He_n$ complexes and Cs_3^+, which increase the yield of Cs_2^+, and the dissociation of bare Cs_2^+, which decreases the yield.

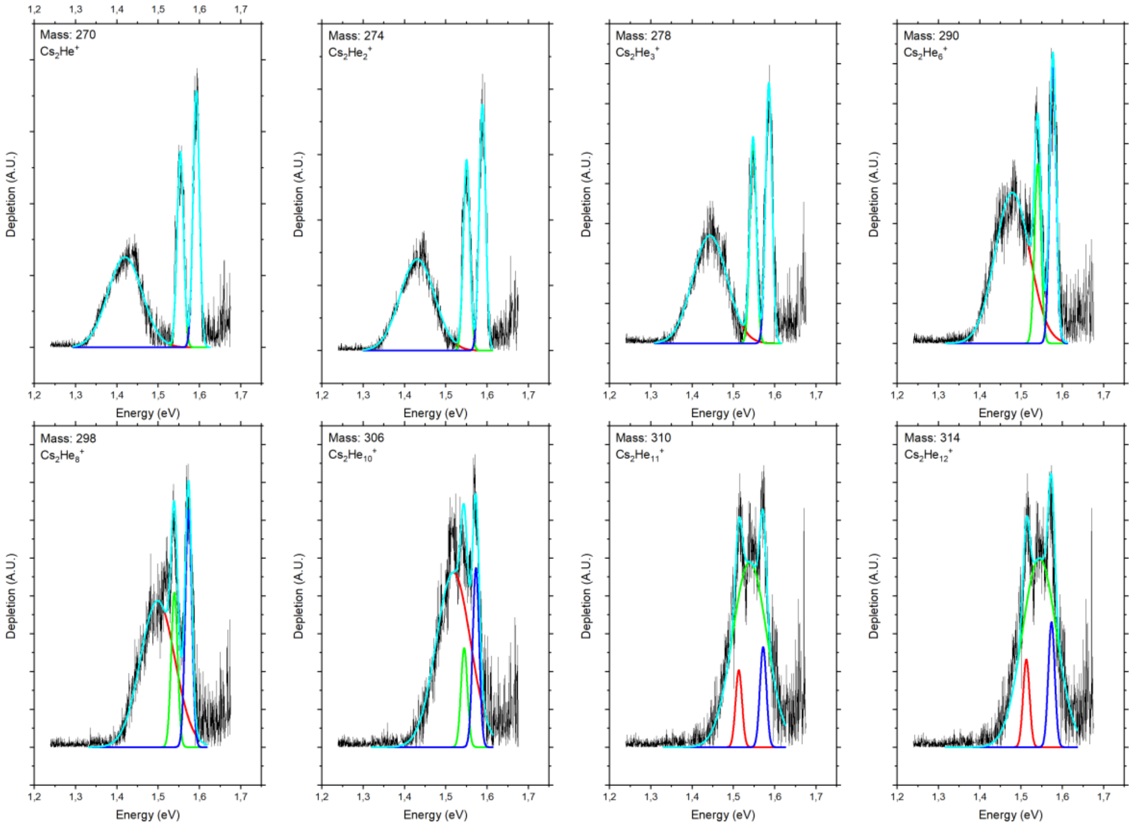

Figure S12: The corrected depletion spectra in the 1.2–1.7 eV region fitted with multi-Gaussian peaks.

6. Cartesian coordinates of $Cs_2^+He_n$ clusters optimized at the CCSD/def2-QZVP(Cs),def2-TZVP(He) level of theory unless stated otherwise (in Ångstrom, energy in Hartree)

Cs_2^+
E=-39.607616

Cs -0.000000 -0.000000 2.873726
Cs 0.000000 -0.000000 -2.873726

Ia
E=-42.500687

Cs 0.000000 0.000000 3.007012
Cs 0.000000 0.000000 -2.739784
He 0.000000 0.000000 -7.348758

IIa
E=-45.393757

Cs 0.000000 0.000000 2.873077
He 0.000000 0.000000 7.484283
Cs 0.000000 0.000000 -2.873077
He 0.000000 0.000000 -7.484283

IIb
E=-45.393744

Cs 2.631313 -0.107174 -0.000057
Cs -3.113182 0.036931 0.000025
He 6.091986 2.728730 0.000147
He 7.159404 -0.797051 0.000746

IIIa
E=-48.286813

Cs 0.040573 2.766142 0.000000
He -2.894156 6.146461 0.000000
He 0.599691 7.315067 0.000000
Cs 0.040573 -2.979513 -0.000000
He 0.062936 -7.593823 -0.000000

IIIb
E=-48.286786

Cs 2.526895 -0.143750 -0.000061
He 6.952013 -1.162068 -0.000862
He 5.995064 1.874967 -1.905281
He 5.995418 1.872405 1.907208
Cs -3.215713 0.049739 0.000022

IVa
E=-51.179868

He 7.419288 -0.673558 0.002307
He 6.279688 2.831867 -0.006859
Cs 2.872691 -0.078752 0.000401
Cs -2.872417 -0.077562 -0.000496
He -7.417469 -0.681912 -0.000163
He -6.289059 2.822264 0.007327

IVb
E=-51.179856

Cs -0.058495 -3.082091 0.000000
He -0.091153 -7.699830 0.000000
Cs -0.058495 2.663123 0.000000
He -0.889783 7.130529 0.000000
He 2.099080 6.045451 1.909570
He 2.099080 6.045451 -1.909570

IVc
E=-51.179825

Cs 3.304454 0.000019 0.047362
Cs -2.437994 -0.000056 -0.140523
He -5.545649 -3.104481 0.464644
He -6.872288 0.000491 -0.999179
He -5.545372 3.104515 0.464317
He -5.864341 0.000475 2.632141

Va
E=-54.072912

Cs -2.768451 -0.119687 -0.013397
Cs 2.975924 -0.066951 0.005212
He 7.527862 -0.641985 0.033939
He 6.376764 2.856689 -0.048537
He -7.221035 -1.029234 -0.109257
He -6.189763 2.151487 -1.722882
He -6.199331 1.795602 2.071826

Vb
E=-54.072893

He 0.663646 5.661842 3.100016
He 2.838761 5.893854 -0.000000
Cs -0.060516 2.573037 -0.000000
He 0.663646 5.661842 -3.100016
He -0.741463 7.041384 -0.000000
Cs -0.060516 -3.171822 0.000000
He -0.096194 -7.792345 0.000000

Vc
E=-54.072875

Cs 0.000000 0.000000 -2.381100
Cs 0.000000 0.000000 3.363905
He 0.000000 3.223645 -5.405430
He 3.065869 0.996161 -5.405430
He 1.894811 -2.607984 -5.405430
He -1.894811 -2.607984 -5.405430
He -3.065869 0.996161 -5.405430

VIa
E=-56.965956

Cs 2.868135 0.150654 -0.037600
He 7.273253 1.238742 -0.362705
He 6.365184 -0.959989 2.483632
He 6.435232 -2.298428 -1.081074
Cs -2.868131 -0.150446 -0.038399
He -7.273361 -1.234496 -0.375464
He -6.367216 0.939387 2.489241
He -6.433219 2.309067 -1.063667

VIb
E=-56.965950

Cs 2.678145 -0.047070 -0.113195
He 5.754309 3.046838 -0.937213
He 7.134218 -0.361517 -0.814550
He 6.055163 1.268951 2.404597
Cs -3.065431 -0.069183 -0.026653
He -6.482899 2.318450 1.636756
He -7.617394 -0.587642 -0.320976
He 5.806969 -2.488123 1.877220

VIc
E=-56.965945

Cs -2.511573 -0.000349 -0.000812
Cs 3.232756 0.000027 0.000071
He -5.523057 -3.237802 0.069199
He -5.526372 2.657588 1.841196
He -5.556578 -1.059178 -3.027085
He -5.535183 2.580633 -1.939946
He 7.857122 0.000257 0.000702
He -5.548463 -0.932619 3.076310

VId
E=-56.965894

Cs -2.277626 -0.000009 -0.000009
He -5.189240 1.461002 -2.948387
He -6.767204 -0.000059 -0.000015
He -5.189779 3.255577 0.478203
He -5.189448 0.551321 3.243968
He -5.189601 -2.352877 -2.300458
Cs 3.467252 0.000004 0.000004
He -5.189440 -2.914844 1.526801

VIIIa
E=-62.752031

Cs 2.867599 -0.009208 0.152589
Cs -2.867631 -0.009657 -0.152613
He 6.390247 0.238350 -2.504221
He 7.289053 -0.078576 1.133318
He 6.023718 -3.042124 -0.628867
He 5.999831 3.138081 -0.083735
He -6.025295 -3.040129 0.630086
He -5.997899 3.139334 0.081703
He -7.289361 -0.077603 -1.132721
He -6.389420 0.241454 2.505098

VIIIb
E=-62.752045

Cs 0.048426 -2.720619 0.000000
Cs 0.048426 3.022531 0.000000
He -0.957944 -5.758548 3.060371
He -3.183765 -5.747991 0.000000
He -0.957944 -5.758548 -3.060371
He 2.643731 -5.769228 -1.891709
He 2.643731 -5.769228 1.891709
He -1.964027 6.522616 1.900652
He 1.076826 7.455728 0.000000
He -1.964027 6.522616 -1.900652

VIIIc
E=-62.752019

Cs -3.228698 -0.100161 -0.000392
Cs 2.513768 0.013657 0.000295
He 5.369267 3.255434 0.825811
He 5.425569 0.268304 3.286596
He 5.493406 -2.989173 1.202421
He 5.474346 -2.014244 -2.541869
He 5.392618 1.845898 -2.774463
He 7.008492 0.103865 -0.000915
He -6.731291 2.717408 0.008143
He -7.771819 -0.808633 -0.003062

VIIId
E=-62.751985

Cs -3.400817 0.000076 0.000015
Cs 2.343243 0.000053 0.000200
He 5.048445 3.209569 -1.435885
He 5.070862 2.833858 2.048399
He 5.056761 -0.359459 3.490153
He 5.063089 -3.194238 1.429621
He 5.047862 -2.849672 -2.058574
He 5.071014 0.357095 -3.476515
He 6.755012 -0.000737 -0.002759
He -8.029754 0.000056 -0.000337

VIIIe
E=-62.751904

Cs 2.148968 0.000025 -0.000053
Cs -3.595090 -0.000036 0.000007
He 6.348496 -1.665814 -0.000604
He 5.151871 0.000789 3.175298
He 4.192825 -3.449936 1.977632
He 4.191517 -3.450999 -1.975583
He 5.152894 -0.000288 -3.175178
He 4.191979 3.449597 -1.977406
He 4.190890 3.451509 1.975886
He 6.347893 1.665461 0.001206

Xa
E=-68.538132
Cs 0.000000 0.000000 2.870994
Cs 0.000000 0.000000 -2.870994
He 0.000000 3.212518 -5.929981
He 3.055286 0.992723 -5.929981
He 1.888270 -2.598981 -5.929981
He -1.888270 -2.598981 -5.929981
He -3.055286 0.992723 -5.929981
He -0.000000 3.212518 5.929981
He -3.055286 0.992723 5.929981
He -1.888270 -2.598981 5.929981
He 1.888270 -2.598981 5.929981
He 3.055286 0.992723 5.929981

Xb
E=-68.538100

He 6.507669 0.004233 2.615407
He 7.479469 -0.001795 -1.020797
Cs 3.034537 -0.000213 -0.129915
He 6.178807 -3.098840 0.457170
He 6.177261 3.101182 0.452193
Cs -2.706131 0.000002 0.016819
He -5.619149 -3.235112 0.684510
He -5.546595 -0.438509 3.353985
He -5.596865 2.965226 1.518148
He -5.695994 2.269990 -2.285494
He -5.709690 -1.561428 -2.799818
He -7.206082 0.000856 0.134825

Xc
E=-68.538083

Cs 3.190400 -0.135030 -0.001112
Cs -2.550415 0.017605 -0.000092
He 7.628976 -1.180130 -0.014351
He -5.256782 1.098911 -3.348111
He 6.701591 1.841963 1.922768
He 6.699498 1.881379 -1.886572
He -5.223070 2.484697 2.546547
He -5.179178 3.507747 -0.803956
He -5.377826 -3.308484 0.801606
He -6.965640 0.134589 0.003201
He -5.336951 -2.311688 -2.555637
He -5.290194 -0.919803 3.367618

Xd
E=-68.538029

Cs 2.381539 0.004734 0.010250
Cs -3.360099 -0.041902 -0.092427
He 4.411568 -3.006765 2.622285
He 4.451484 3.054794 -2.527063
He 4.376400 3.774333 1.361440
He 5.326106 0.610163 3.187938
He 6.594346 -1.595639 0.390550
He 5.455501 -0.548207 -3.052646
He 4.488317 -3.732214 -1.262689
He 6.577169 1.678431 -0.217299
He -6.861594 1.114790 2.488832
He -7.908887 -0.327569 -0.731491

Xe
E=-68.537982

He -4.039366 -3.766218 1.801329
He -5.230945 -0.725593 3.153005
He -6.348064 -1.815022 0.003748
He -4.050717 -3.728607 -1.859133
He -6.318666 1.621648 0.008425
He -4.341681 2.598625 -3.008451
He -3.951481 4.249202 0.023451
He -4.353340 2.557437 3.030098
He -5.261818 -0.670022 -3.137961
Cs -2.221637 -0.008911 -0.000473
Cs 3.521312 -0.002920 -0.000061
He 8.155035 0.003904 0.000161

Xf
E=-68.537906

He -4.333184 1.026180 3.641901
He -6.095498 -1.133690 1.684222
Cs -2.038163 -0.009831 -0.000009
He -4.307195 -3.798113 0.010134
He -6.095696 -1.136890 -1.679269
He -4.329447 1.004855 -3.651859
He -3.722632 3.884463 1.774698
He -3.728064 3.870550 -1.795220
He -5.997025 1.943731 -0.000706
Cs 3.704833 -0.004374 0.000022
He -3.613249 -2.645231 -3.428389
He -3.611429 -2.625220 3.444133

XIIa
E=-74.324166

Cs 0.000000 0.000000 2.870828
Cs 0.000000 0.000000 -2.870828
He 0.000000 0.000000 7.378795
He 0.000000 0.000000 -7.378795
He 0.000000 3.289586 5.808546
He -3.128583 1.016538 5.808546
He -1.933570 -2.661331 5.808546
He 1.933570 -2.661331 5.808546
He 3.128583 1.016538 5.808546
He 1.933570 2.661331 -5.808546

He 3.128583 -1.016538 -5.808546
He -0.000000 -3.289586 -5.808546
He -3.128583 -1.016538 -5.808546
He -1.933570 2.661331 -5.808546

XIIb
E=-74.324172

Cs -3.039570 0.000047 -0.000032
He -6.100281 2.435365 -2.102067
He -6.099243 -1.247380 -2.966989
He -6.099386 2.752499 1.667628
He -6.099635 -0.734950 3.133564
He -6.100691 -3.206356 0.269321
Cs 2.702003 0.000013 -0.000295
He 5.441663 -0.169249 3.499809
He 5.443674 2.944997 1.895359
He 5.439659 3.119011 -1.603482
He 5.442526 -3.114878 1.603455
He 5.440722 -2.949336 -1.895673
He 7.125096 -0.000252 0.001293
He 5.448991 0.168861 -3.493230

XIIc
E=-74.324110

Cs 2.571969 0.008980 0.011941
Cs -3.167743 -0.075439 -0.110314
He 4.525564 2.769696 2.950834
He 6.816102 -1.521562 -0.379690
He 4.733405 -2.689963 -2.837612
He 5.537261 -0.866212 3.114395
He 4.669477 -3.837482 0.950194
He 5.643517 0.975848 -2.956209
He 6.748508 1.666410 0.587825
He 4.593013 3.917731 -0.834236
He -6.638018 1.470615 2.173231
He -6.308562 -2.310956 2.129986
He -6.315260 2.824367 -1.361729
He -7.621227 -0.570852 -0.831733

XIId
E=-74.324082

Cs 2.426847 0.006665 0.001062
He 4.489892 2.656793 3.018117
He 4.042917 4.317435 -0.002329
He 5.459288 -0.599221 3.162612
He 4.347955 -3.688422 1.840472
He 4.369414 -3.677202 -1.830832
He 5.503850 -0.590788 -3.120391
He 6.483011 1.751529 0.008643
He 4.492020 2.654051 -3.020820
He 6.601529 -1.689804 0.027582
Cs -3.312980 -0.138592 -0.012905
He -6.834491 1.655855 2.076423
He -6.834275 2.027729 -1.715940
He -7.752432 -1.189950 -0.117857

XIIe
E=-74.324032

Cs -2.266499 0.018884 0.001199
Cs 3.473880 -0.100055 0.021580
He -4.628632 -2.416873 2.851641
He -4.042788 -4.227293 -0.057938
He -6.331310 0.408181 1.990029
He -3.843873 0.953862 4.230851
He -6.288449 1.818200 -1.068265
He -3.765167 3.907252 -1.966973
He -4.510213 0.733023 -3.760255
He -4.035629 -2.666903 -3.275760
He -6.270666 -1.672359 -0.820819
He -4.486984 3.480341 1.613291
He 6.971493 2.691268 -0.508350
He 8.029237 -0.776503 0.146105

XIIf
E=-74.323945

Cs -2.114086 -0.000534 -0.004192
Cs 3.627317 -0.000068 -0.005181
He -4.067551 -1.940414 -3.506852
He -6.380144 0.005125 -1.885593
He -5.678822 2.734418 -0.216226
He -3.101167 4.657035 -0.933428
He -5.685011 -2.727750 -0.219893
He -3.109415 -4.649773 -0.948649
He -4.063621 1.953753 -3.501615
He -3.957541 3.134716 2.651868
He -3.957340 -3.143800 2.641951
He -6.134706 0.001232 1.876788
He -3.744175 -0.008209 4.300972
He 8.265617 0.000201 -0.001541

XIIg
E=-74.323878

He 3.827478 2.922653 2.892779
He 5.641380 2.578201 0.006468
Cs 1.947910 -0.000499 -0.000230
He 5.833879 0.005668 -2.158916
He 3.271239 0.013557 -4.576017
He 2.976684 -4.767555 -0.009867
He 5.831830 -0.004950 2.159303
He 3.827320 -2.934645 2.882634
He 5.643116 -2.576410 -0.004673
Cs -3.793573 0.000027 0.000015
He 3.828878 -2.921389 -2.894359
He 2.973333 4.769620 0.012165
He 3.271807 -0.008403 4.577311
He 3.828789 2.936642 -2.880929

Cs_2^+, CCSD/def2QZVPPD
Cs -0.000000 0.000000 2.865225
Cs 0.000000 0.000000 -2.865225

Cs_2^+, CCSD/def2QZVPPD, $1^2\Pi_u$
Cs 0.000000 0.000000 2.894314
Cs 0.000000 0.000000 -2.894314

Cs_2He^+, CCSD/def2QZVPPD

Cs	0.000000	0.000000	2.995631
Cs	0.000000	0.000000	-2.735122
He	0.000000	0.000000	-7.163973

Cs_2He^+, CCSD/def2QZVPPD, $1^2\Pi_u$
Cs 0.029816 -3.010484 0.000000
Cs 0.029816 2.779227 0.000000
He -1.639899 6.359586 0.000000
